# Low Prevalence of *Leptospira* Carriage in Rodents in Leptospirosis-Endemic Northeastern Thailand

**DOI:** 10.3390/tropicalmed5040154

**Published:** 2020-09-30

**Authors:** Panadda Krairojananan, Janjira Thaipadungpanit, Surachai Leepitakrat, Taweesak Monkanna, Elizabeth W. Wanja, Anthony L. Schuster, Federico Costa, B. Katherine Poole-Smith, Patrick W. McCardle

**Affiliations:** 1 Department of Entomology, Armed Forces Research Institute of Medical Sciences (AFRIMS), Bangkok 10400, Thailand; SurachaiL.fsn@afrims.org (S.L.); TaweesakM.fsn@afrims.org (T.M.); Betty.Poolesmith.mil@afrims.org (B.K.P.-S.); Patrick.McCardle.mil@afrims.org (P.W.M.); 2 Department of Clinical Tropical Medicine, Faculty of Tropical Medicine, Mahidol University, Bangkok 10400, Thailand; janjira.tha@mahidol.ac.th; 3 Mahidol-Oxford Tropical Medicine Research Unit, Faculty of Tropical Medicine, Mahidol University, Bangkok 10400, Thailand; 4 Department of Preventive Medicine and Biostatistics, Uniformed Services University of the Health Sciences, 4301 Jones Bridge Road, Bethesda, MD 20814, USA; Elizabeth.wanja@usuhs.edu; 5 MEDCOM Headquarters, Fort Sam Houston, San Antonio, TX 78234, USA; anthony.l.schuster.civ@mail.mil; 6 The Institute of Collective Health (ISC), Federal University of Bahia (UFBA), Rua Basílio da Gama, 316, Canela, Salvador CEP 40110-040, Brazil; frederico.costa@yale.edu; 7 Department of Epidemiology of Microbial Diseases, Yale School of Public Health, 60 College St, New Haven, CT 06510, USA

**Keywords:** carriage, *Leptospira*, *L*. *wolffii*, rodent, *Bandicotaindica*, 16S rRNA gene, Thailand

## Abstract

Leptospirosis is a neglected zoonotic disease affecting mostly the world’s tropical regions. The rural people of northeastern Thailand suffer from a large number of leptospirosis infections, and their abundant rice fields are optimal rodent habitats. To evaluate the contribution of diversity and carriage rate of pathogenic *Leptospira* in rodent reservoirs to leptospirosis incidence, we surveyed rodents, between 2011 and 2012, in four provinces in northeastern Thailand with the highest incidence rates of human leptospirosis cases. We used *lipL32* real-time PCR to detect pathogenic *Leptospira* in rodent kidneys, partial 16S rRNA gene sequencing to classify the infecting *Leptospira* species, and whole 16S rDNA sequencing to classify species of isolated *Leptospira*. Overall prevalence of *Leptospira* infection was 3.6% (18/495). Among infected rodents, *Bandicota* *indica* (14.3%), *Rattus* *exulans* (3.6%), and *R*. *rattus* (3.2%) had renal carriage. We identified two pathogenic *Leptospira* species: *L*. *interrogans* (*n* = 15) and *L*. *borgpetersenii* (*n* = 3). In addition, an *L*. *wolffii* (LS0914U) isolate was recovered from the urine of *B*. *indica*. *Leptospira* infection was more prevalent in low density rodent populations, such as *B*. *indica*. In contrast, there was a lower prevalence of *Leptospira* infection in high density rodent populations of *R*. *exulans* and *R*. *rattus*.

## 1. Introduction

Pathogenic *Leptospira* species are the causative agents of leptospirosis, one of the most common zoonotic diseases in the world [1]. Although rodents are the primary reservoir hosts for *Leptospira*, a variety of domestic and wild animals, such as dogs, buffaloes, cattle, pigs, and mongooses also serve as reservoirs and may transmit the disease to other animals through direct and indirect exposure to infected urine [2,3]. Infected animals shed *Leptospira* from their renal tubules into the environment via urine [4,5]. Most human leptospirosis cases are the result of contact with water or soil contaminated with the urine of infected animals [1].

In Thailand, leptospirosis is a reportable disease under the passive surveillance system of the Bureau of Epidemiology, Department of Disease Control, Ministry of Public Health (MoPH). The vast majority of human cases in northeastern Thailand occur during the rainy season (July to October). Agricultural workers, primarily rice farmers, are at higher risk, due to traditional practices of barefoot rice farming [6]. Rodent populations in rice fields have been reported as a potential sources of *Leptospira*. In previous studies, the seroprevalence rates of *Leptospira* in rodents trapped during 1998 to 2000 in epidemic areas from 10 provinces (Burirum, Udon Thani, Nakhon Ratchasima, Phetchabun, Phitsanulok, Chanthaburi, Chon Buri, Phra Nakhon Si Ayutthaya, Trang, and Surat Thani) across Thailand was approximately 4.8%, with most seropositive rodents being found in northeastern rodent populations (7.1%) [7].

The prevalence of Leptospirosis is underestimated. One reason for the underreporting of leptospirosis cases is that its nonspecific symptoms make it difficult to distinguish from other tropical diseases such as dengue, scrub typhus, and malaria [8,9]. Exacerbating this problem, Thailand lacks a system linking laboratory results back into the MoPH surveillance system, so leptospirosis surveillance is based on presumptive cases rather than laboratory confirmed cases [10]. Between 2006–2018, most leptospirosis cases (65–83%) in the northeastern region of Thailand reported to the Thai MoPH were in populations working in agriculture-related jobs. While 47 to 64% of leptospirosis cases reported nationally in Thailand’s MoPH passive surveillance system were attributed to occupational exposure among farmers [11], epidemiological studies showed that peridomestic environments contribute significantly to the leptospirosis burden in Thailand [7,12]. Moreover, little is known about which *Leptospira* strains normally circulate in northeastern Thailand. This lack of knowledge makes it difficult to attribute human disease to leptospirosis prevalence in specific rodent species. Understanding this relationship is critical in deciphering disease epidemiology and may be useful for leptospirosis prevention.

Previously, our preliminary survey data suggested a pattern of low prevalence of *Leptospira* in rodents related with a high incidence of human leptospirosis (unpublished data). In this study, we evaluated *Leptospira* prevalence in rodents, between 2011 and 2012, from four provinces in the northeastern region of Thailand with the highest incidence rates of human leptospirosis cases: Burirum, Kalasin, Sisaket, and Surin.

## 2. Materials and Methods

### 2.1. Trapping Rodents

**Figure 1:**
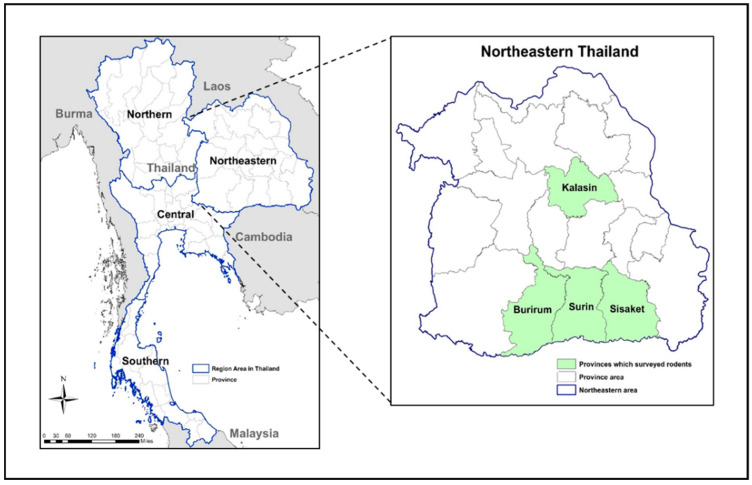
Map of Thailand showing the provincial locations for pathogenic *Leptospira* surveillance in rodent populations between 2011 and 2012. Four provinces (Burirum, Kalasin, Sisaket, and Surin) located in the northeast region of Thailand.

Rodents were trapped in the Buriram, Kalasin, Sisaket, and Surin provinces in the northeastern region of Thailand (Figure 1). Rodent trapping was carried out only in areas that had reported human leptospirosis cases. Clusters of two or three villages were sampled during each trapping session. Each session lasted three consecutive nights and used 100 small wire live traps per night. In areas where reported cases were close together, three villages were sampled, while only two villages were sampled in areas where villages were far apart. In each trapping site, traps were randomly distributed and placed 5 m apart. To avoid heat stress for the rodents, trap placement was completed before 6 p.m. in the evening and animals were collected before 7 a.m. the following morning. Individual traps were baited with bananas or dried fish and then placed in different habitats in relation to human activity, including with houses (in the garden), flood plains, dry lands, and forested areas. To determine the relative abundance of the trapped rodents, trap success (TS) was calculated as described by Nelson and Clark [13]: TS (%) = (Tc/Tn) × 100, where TS = trapping success, Tc = the number of rodents trapped and Tn = the number of trap nights. The trap nights were calculated by multiplying the number of fully functional traps by the number of trap nights. Each morning trap was inspected and the number of trapped rodents counted.

### 2.2. Rodent Identification and Processing

Trapped rodents were euthanized with carbon dioxide and identified at a species level. Species identification was based on external morphological guidelines by Lekagul and McNeely [14]. Necropsy was performed and tissues were collected. Direct puncture of a full urinary bladder was performed under aseptic conditions for urine collection (when available, 13 urine samples/495 rodents) for culture. One kidney from each rodent was removed to culture and isolate *Leptospira*. All procedures involving animals were in accordance with animal use protocols approved by the Institutional Animal Care and Use Committee (IACUC) of the Armed Forces Research Institute of Medical Science (AFRIMS), Bangkok, Thailand (Protocol Number 12-06, Field Sampling of Small Mammal; Orders: Erinaceomorpha; Soricomorpha; Scandentia; Macroscelidea and Rodentia; Populations to Support Zoonotic Diseases Surveillance and Ectoparasite Collection).

### 2.3. Leptospira Cultures

At the field site, all culturing was performed using 5-fluorouracil-containing Ellinghausen-McCullough-Johnson-Harris (EMJH) medium (Difco, Franklin Lakes, NJ, USA) with 5% rabbit serum. Each urine sample was dropped in 3 mL of 0.1% semisolid EMJH medium. A section of the cortex from one kidney of each animal was homogenized by forcing tissue through a disposable 3 mL syringe attached to 18-G hypodermic needle. Homogenates were suspended in 5 mL culture EMJH medium and tissue debris was allowed to precipitate. After 24 h, 500 µL of the culture was subcultured into 3 mL semisolid EMJH medium and stored at room temperature until being transported back to AFRIMS. At the AFRIMS laboratory, the EMJH culture vials were incubated for 16 weeks at 30 °C with biweekly dark field microscopic observation to verify the presence of *Leptospira*.

### 2.4. Leptospira Carriage and Molecular Identification in Trapped Rodents

Genomic DNA was extracted from kidney specimens using Wizard^®^Genomic DNA Purification Kit (Promega, Madison, WI, USA) according to the manufacturer’s instructions. The presence of *Leptospira* DNA was screened using the *lipL32* real-time assay that was modified from McAvin et al. [15] to suit the Chromo4™ System (Bio-Rad, Hercules, CA, USA) as follows: 25 µL of reaction mixtures were combined with 1 × RealMasterMix Probe (0.04 units/µL HotMaster Taq DNA Polymerase, 2 mM Magnesium Acetate, 0.4 mM dNTPs with dUTP) (5 PRIME, Gaithersburg, MD, USA) according to manufacturer’s instructions, with 0.1 µM probe LPS-TM and 0.2 µM each of primers LPS-F and LPS-R. Positive and negative control samples were included in each round of testing. The positive controls included DNA prepared from reference *L*. *interrogans* culture and from *Leptospira*-infected rodent kidney tissues collected during previous surveillance studies. DNA prepared from nonpathogenic *L*. *biflexa* culture and from noninfected rodent tissue were used as negative controls in the assay. Real-time PCR was performed under the following conditions: 95 °C for 1 min, 45 cycles at 95 °C for 15 s, 60 °C for 30 s. Test samples giving cycle-to-threshold (Ct) values lower than 40 were analyzed as positive. A nested single tube PCR assay [16] targeting partial 16S rDNA was performed on positive *lipL32* real-time assay samples. Amplicons were visualized using 1.5% gel electrophoresis followed by staining with GelStar (Lonza, Basel, Switzerland). Positive PCR samples were purified by GeneJET^TM^PCR Purification Kit (Fermentas, Waltham, MA, USA). After purification, samples were sent to the Macrogen laboratory (Macrogen Inc., Seoul, Korea) for Sanger sequencing. Molecular identification of *Leptospira* species by DNA sequencing of partial 16S rDNA amplicon from each DNA extracted from rodent’s kidney samples was aligned using Sequencher^TM^v5.0 software (Gene Codes Corporation, Miami, FL, USA), and trimmed to the 443 base-pairs region. Maximum likelihood trees were reconstructed from partial 16S rDNA sequence using an algorithm implemented in MEGA X version 10.0.5 [17]. The model of sequence evolution used was the generalized time-reversible (GTR) model with gamma-distributed rate variation. The model parameters were adjusted as follows: transition/transversion ratio was fixed to 4.0, and the gamma shape parameter accounting for rate variation among sites and the proportion of invariant sites was optimized. We set the program to search the tree using the Nearest Neighbor Interchange (NNI) method and chose BioNJ as an initial tree. Interactive Tree of Life (iTOL) v4 [18] was used to display and edit the tree.

### 2.5. 16S rRNA Gene Amplification and Sequencing Analysis of Recovered Leptospira Isolate

The isolated *Leptospira* was grown in liquid EMJH and incubated at 30 °C for 7 days. Total genomic DNA was extracted from the culture using the GeneJET^TM^Genomic DNA Purification kit according to manufacturer’s instructions (Fermentas, Waltham, MA, USA). PCR was performed using the 16S rRNA primer fD1/rD1 [19]. After the first round of amplification, PCR products were diluted to 1:10^3^, followed by a second round of amplification using the nested primers lepto16S11f/lepto16S1338r [20]. A reaction volume of 50 µL was performed using 1 unit of KOD-Plus DNA polymerase (TOYOBO, Japan) with final primer concentration of 300 nM. Amplification was performed using a Veriti^TM^Thermal Cycle (Applied Biosystems, Waltham, MA, USA) with the following protocol: 2 min at 94 °C; 35 cycles 92 °C for 15 s, 63 °C for 30 s, 68 °C for 2 min, then a final extension of 68 °C for 5 min. PCR products were purified from 1% agarose gels in Tris-acetate-EDTA buffer using Quantum Prep^TM^Freeze’N Squeeze DNA Gel Extraction Spin Columns (Bio-Rad, Hercules, CA, USA) according to manufacturer’s instructions. Purified PCR products were sequenced using primers lepto16S11f/lepto16S505f and lepto16S1006f as previously described [20]. Alignment of DNA sequence of the whole 16S rRNA gene and phylogenetic analysis was conducted as described above.

### 2.6. Statistical Analysis

The statistical analysis in this study performed with IBM SPSS Statistics 26.0 (IBM SPSS Statistics for Windows, Version 26.0., IBM Corporation, Armonk, NY, USA). A *p*-value of less than 0.05 was considered statistically significant. Fisher’s exact test was used to compare the association between *Leptospira* prevalence and trapped rodent species and *Leptospira interrogans* prevalence among the *Leptospira*-positive rodents. Fisher’s exact test with Spearman’s rho correlation was done to determine whether correlation existed between human incidence and general *Leptospira* rodent prevalence.

## 3. Results

A total of 495 rodents were trapped from the four provinces reporting the highest incidence of human leptospirosis cases to the MoPH since 2007 (Figure 1). Ten rodent species were trapped and identified (Table 1) based on taxonomic features. Overall trap success was 17.4%. Among trapped rodents, *R. exulans* and *R. rattus* were the most frequently trapped species (87.5%); these species were found in all four provinces. Of the total trapped rodents, 320 (64%) were trapped from Burirum, 84 (17%) from Sisaket, 48 (10%) from Kalasin, and 43 (9%) from Surin.

The overall percentage of pathogenic *Leptospira* infection in kidneys detected by the *lipL32* real time assay was 3.6 (18/495). *Leptospira* prevalence in rodents from each province was determined; the highest infection rates was found in Sisaket (7.1%), followed by Burirum (3.4%) and Surin (2.3%) (Table 1). No rodents from Kalasin were positive for *Leptospira* infection. We observed a positive but not statistically significant correlation between human incidence and general *Leptospira* rodent prevalence (r = 0.4, *p* = 0.6).

**Figure 2:**
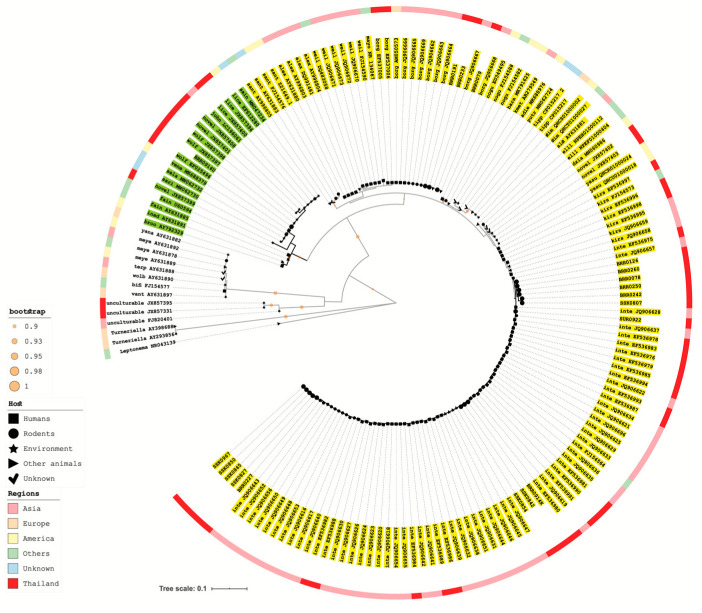
A phylogenetic tree of *Leptospira* species. A maximum likelihood tree was reconstructed from the 443 bp of 16S rDNA sequences using MEGA X version 10.0.5., containing a total of 19 sequences from rodent samples (big black dots) together with 146 reference sequences from GenBank data; this included 36 official Leptospira species, unculturable Leptospira (clustering with the nonpathogen Leptospira species) and probable novel Leptospira species, which were used to reconstruct the tree as shown in Table S1. Three isolates from genus Leptonema and Turneriella were also included as out-groups. Different colors of the highlighted text represent three groups in the genus Leptospira; yellow for species in the pathogen group, green for species in the intermediate group, no highlight for the nonpathogens and out-group. Hosts of isolates are shown in different shapes: dots for rodents, squares for human, triangles for other animals, stars for environmental samples, and checkmarks for unknown hosts. The outer color strip demonstrates the geographical origins of isolates at the continent level (except for isolates from Thailand). Abbreviations are exemplified by the following: BRR, samples from Burirum; SUR, samples from Surin; SSK, samples from Sisaket; BRR0914K, the partial 16S rDNA sequences of the kidney sample from rodent no. 0914 trapped in Burirum; BRR0914U, the partial 16S rDNA sequences from the Leptospira isolated from a urine sample from rodent no. 0914 trapped in Burirum. GenBank accession numbers of the 16S rDNA sequence are provided in Table S1.

By the PCR-based investigation of kidney samples, three species (*B. indica* at 14.3%, *R. exulans* at 3.6%, and *R. rattus* at 3.2%, as shown in Table 1 and Table 2) were identified as *Leptospira* carriers. General *Leptospira* prevalence was higher in *B. indica* when compared to *R. rattus* (*p* = 0.05). *Leptospira interrogans* prevalence was also higher in *B. indica* when compared to *R. exulans* (*p* = 0.03) and *R. rattus* (*p* = 0.05). The *lipL32* PCR assay positivity indicated pathogenic *Leptospira* carriage in rodents. Using the partial 16S rRNA gene sequence analysis and the maximum likelihood phylogenetic tree construction, two pathogenic *Leptospira* species were assigned for these samples: *L. interrogans* (83%) and *L. borgpetersenii* (17%) (Figure 2). The 443- nucleotide sequences of partial 16S rDNA sequences were submitted to GenBank with the accession numbers KP120894-KP120903 and KP120905-KP120912. *L. interrogans* or *L. borgpetersenii* harboring rodents were trapped in Burirum province. *L. interrogans* was found in rodents trapped from Sisaket and Surin (Table 1). *L. borgpetersenii* was only present in *R. exulans*, while *L. interrogans* was found in *B. indica*, *R. exulans*, and *R. rattus*.

None of the kidney cultures from the 495-trapped rodents was positive for *Leptospira*. Urine samples were collected from 13 of these rodents and inoculated for *Leptospira* isolation. Culture was successful for 1/13 urine samples and the near full length of 16S rRNA gene was used to determine the *Leptospira* species. The 16S rDNA sequence of the LS0914U isolate (accession numbers KP120904 for 443 bp of 16S rRNA gene and KC662454 for 1304 bp of 16S rRNA gene) was identical to the sequence of a reference *L. wolffii* serovar Korat (GenBank no. EF025496.1) in the intermediate *Leptospira* group (Figure 2 and Supplementary Materials Figure S1).

## 4. Discussion

The objective of our study was to determine whether rodents with chronic *Leptospira* infection in highly leptospirosis endemic northeastern Thailand could be implicated as the source of human *Leptospira* infection. From 2011 to 2012, the MoPH reported annual leptospirosis incidence rates ranging from 16.8–30.9 per 100,000 in Burirum, Kalasin, Sisaket, and Surin provinces, which were higher than the overall average annual incidence of 6.6 per 100,000 for Thailand during the same period.

Three point six percent of rodents trapped in this study were positive for pathogenic *Leptospira* carriage. Most of the rodents, i.e., 87.5%, trapped in this study were *R*. *rattus* and *R*. *exulans.* The rodent species trapped in this study are consistent with those reported in previous leptospirosis studies in Thailand [7,12,21,22]. These rodent species are commonly found in habitat types ranging from field to household, with each species showing distinct preferences for specific habitats within that range [23,24]. *Rattus* spp. were the most abundant species (15.0%) and had a lower *Leptospira* prevalence (3.5%) than other trapped species. These findings are consistent with the results from a study by Ivanova in Cambodia [24]. These more populous rodent species were trapped around households and in drier habitats which could be less suitable for *Leptospira* growth. The highest prevalence of *Leptospira* infection (14.3%) was in rodents with the lowest density populations, i.e., *B. indica* (0.7%), which suggests that this rodent species may play a relevant role in human *Leptospira* transmission in Thailand. In fact, during a leptospirosis outbreak in 2000, in the same region, a pathogenic clone of *L. interrogans* serovar Autumnalis ST34 was predominantly found in bandicoot rats and human cases [25]. Another survey of *Leptospira* infection in rodents in northern Thailand also reported high leptospirosis prevalence (10.7%) in *B. indica* [21]. Moreover, habitat preference studies on *B. indica* have indicated that this species prefers agricultural fields [26,27], a prime environment for survival of *Leptospira* [28,29], and is rarely reported around or inside houses [26]. *Leptospira* prevalence in *B*. *indica* was highly variable between sites (0–50%). However, the sample size was low for this species, given that our study was performed in villages, which is not the primary habitat for *B. indica*.

Despite the variety of rodent species captured, we detected only two *Leptospira* species, *L*. *interrogans* and *L*. *borgpetersenii*. A large proportion of rodent species were positive to *L*. *interrogans* (83%; 15 of 18) (Figure S1). Assuming that this infection rate is representative of *Leptospira* infections in rodents, we would expect more environmental contamination with this *Leptospira* species compared to other species. Our observation of higher prevalence of *L*. *interrogans* than *L*. *borgpetersenii* in rodent population is consistent with previous work (unpublished data). The contribution of *L*. *interrogans* to human transmission in Thailand has been reported in previous studies, and was responsible for 96% of leptospirosis cases [25]. The ability of *L*. *interrogans* to survive longer in the environment compared to *L*. *borgpetersenii* may increase the likelihood of transmission from rodents to humans [30]. In our study, *L*. *borgpetersenii* was only identified in *R*. *exulans*. *Rattus exulans* were mainly captured around human residences. A study by Cosson et al. [21] on the effects of host species, sex, maturity, habitat, and locality on infection status found that the rodent locality, ecological habitat, and sex significantly influenced the likelihood of individual rodent infection. Moreover, their study also showed that the likelihood of *Leptospira* infection was not related to specific rodent species, but rather, to whether the habitat that the rodents were collected in was suitable for *Leptospira* survival. *Leptospira borgpetersenii* requires host to host transmission, and thus, can be transmitted in dry, peridomestic habitats [30], whereas *L*. *interrogans* requires humidity for survival [21]. This difference in survival requirements could explain why even though *R. exulans* can be infected with *L*. *interrogans* or *L*. *borgpetersenii* and the species is found peridomestically, the transmission of *L*. *borgpetersenii* between rodents and humans is less likely. *Leptospira borgpetersenii* requires direct contact for transmission, since it is unable to survive outside a host for prolonged periods. Therefore, transmission of *L*. *borgpetersenii* to humans is less likely, with only 3% of human leptospirosis infections being caused by *L*. *borgpetersenii*, as reported by Thaipadungpanit et al. [25], since direct contact between humans and rodents is rare. Transmission of *L. interrogans* to humans is more frequent, because this strain is able to survive in wet environments, and practices like barefoot rice farming bring humans into prolonged, close contact with such ideal, moist environments. Rodents are attracted to rice fields and, in their search for food, shed *Leptospira* in their urine as they move through the rice fields. Farmers spend hours immersed in water, often barefoot, while tending these rice fields, which increases the likelihood of infection by *L. interrogans* through any open wounds. These findings are in the same vein as those in a previous study reporting that *L*. *interrogans* and *L*. *borgpetersenii* required a difference in ecological niche to support their transmission [21].

In addition to detecting *L*. *interrogans* and *L*. *borgpetersenii*, we also isolated *L*. *wolffii* from one urine sample (Figure S1). The presence of *L*. *wolffii* in the urine culture could be explained by the ease of culturing this species compared to *L*. *interrogans*. The real time-PCR cycle threshold for sample BRR0914 ranged from 35–40, suggesting low number of *Leptospira* copies in the kidneys. The low load may be related to continuous host immune response, as suggested by Monahan et al. [31]. In the presence of a low-level coinfection, selective pressure in the urine culture media could favor the growth of *L*. *wolffii* over *L*. *interrogans*. The *lipL32* gene is specific to pathogenic *Leptospira*. However, there is evidence for the possibility of co-infection with intermediate and nonpathogenic *Leptospira*, which are not detected using *lipL32*. *Leptospira wolffii* is a known environmental contaminant and potential pathogen [32,33,34] which has previously been detected in both animals and clinical human samples [35,36,37,38]. The presence of *L*. *wolffii* in culture was likely due to media competitive exclusion, but its importance in human disease is still inconclusive. Interestingly, an increasing number of human leptospirosis cases caused by the intermediate strains of *Leptospira* have been reported, such as *L*. *wolffii* in Thailand [38], India [36], Iran [35,37], and Ecuador [39], and *Leptospira licerasiae* in the Peruvian Amazon [40,41] and in a Japanese case returning from Brazil [42].

Human infection by pathogenic *Leptospira* can occur along several pathways. Often, activities associated with agricultural work, including cultivation and husbandry, are positively associated with exposure. Even something as common as living in a house with wooden walls can lead to an increased likelihood of infection due to a favorable environment for rodent infestation and residual, infectious nesting [43]. Although rodents were generally plentiful in our study sites, since leptospirosis is a zoonotic disease, the fact that other reservoirs, such as cattle and dogs, were found in the study sites led us to consider nonrodent sources of *Leptospira* transmission. According to a 1999 serosurvey of livestock by Thailand National Institute of Animal Health (NIAH), 69.2% of cattle tested in the northeastern provinces were positive for *Leptospira* [44]. A second NIAH serological investigation in 2001 found that 11.5% of livestock were seropositive for *Leptospira* across 36 Thai provinces. Of the locations surveyed, 30% of the infected animals were in the northeast region [45]. In addition, Wongpanit et al. [46] found a 63.6% *Leptospira* seroprevalence in swamp buffalo in SakhonNakhon, a northeastern province. Farmers in the northeast commonly apply traditional rice farming methods involving the use of buffalo and cattle in paddy rice field preparation. These animals are often free to move between the household and the fields to graze when not being actively used by the farmer. Suwancharoen et al. [47] found that both water buffalo and domestic cattle throughout Thailand were shedding active *Leptospira* in their urine, highlighting another way these pathogens may circulate or be maintained in the environment. Even common domestic activities have a risk associated with this disease. In Nan province, Thailand, pathogenic *L. interrogans* were detected in asymptomatic humans, various household animals (cattle, dogs, and pigs) and a local underground water source often used for animal husbandry, i.e., for drinking or cleaning pens [48].

This study had several limitations: peridomestic sampling, potential sampling bias, and small numbers of non-*Rattus* species collected. We sampled rodents in villages near reported human leptospirosis cases. Traps were placed in areas of highest human activity, resulting in greater numbers of traps placed around housing rather than fields. As a result, our collections may be biased towards more rodents collected around houses than fields. Additionally, we collected small numbers of non-*Rattus* species, i.e., comprising 6% of the total rodents collected. The factors resulting in low collection rates of non-*Rattus* species vary by species, with the larger species *B*. *indica* and *B*. *savilei* being valued for human consumption; therefore, local practices of trapping and eating these species may have been the reason for the relatively lower numbers collected. Additionally, *M*. *cervicolor* and *M*. *caroli* prefer rice and corn fields, while *S*. *murinus* are found inside houses; as such, we may have collected relatively smaller numbers of these three species due to peridomestic sampling bias. *Suncus murinus* rodents are reported to inhabit multiple Southeast Asian countries and to prefer peridomestic habitats; however, two studies of rodent surveillance for leptospirosis in Thailand and Southeast Asia [7,21] did not report collection of this rodent species. In contrast, a survey of Ivanova et al. of rodents in Cambodia reported high numbers of *S*. *murinus* (9% or 57/650), but noted that these were collected only in households. Therefore, either there are lower numbers of *S*. *murinus* in Thailand than Cambodia, or our peridomestic sampling methods may have biased us against collecting this species.

## 5. Conclusions

We detected pathogenic *Leptospira* in *B*. *indica*, *R*. *exulans*, and *R*. *rattus*, the reservoir hosts of *Leptospira* in northeastern Thailand. Two pathogenic *Leptospira*, *L*. *borgpetersenii* and *L*. *interrogans*, were detected in rodent populations. *L*. *wolffii* was found in the urine culture of *B*. *indica*, whose kidney samples were PCR-positive for *L*. *interrogans* and negative in culture. The proximity of reservoir habitats to sites of human activities may have facilitated transmission by exposure to an infected urine-contaminated environment. *L*. *wolffii*, recovered and isolated from rodent urine, could be a potential pathogen surviving in the environment and transmitting among rodents, the environment, and humans. The low evidence of *Leptospira* carriage in rodents from village areas with high incidence of human cases may indicate that other animals, especially domesticated animals, serve as potential reservoirs and play a role as transmission drivers of human leptospirosis. Additionally, the prevalence of carriage in *B. indica* in agricultural settings will be relevant to better understanding the contribution of this species to leptospirosis transmission.

## Figures and Tables

**Table 1:** Number of trapped rodents, PCR positive for *Leptospira* by study area and rodent species relative to the average annual incidence of human cases per 100,000 people reported to the MoPH in 2011 and 2012.

	Average Annual Incidence of Human Cases Per 100,000	Number of Trapped Rodents (%)	Percentage of Trap Success	*Leptospira* Prevalence (%)		
				All	Linte ^1^	Lborg ^1^
**Provinces**						
Burirum	23.54	320 (64.6)	15.72	11 (3.4)	8 (2.5)	3 (0.9)
Surin	29.63	43 (8.7)	15.47	1 (2.3)	1 (2.3)	0
Sisaket	26.97	84 (17.0)	32.85	6 (7.1)	6 (7.1)	0
Kalasin	19.28	48 (9.7)	17.44	0	0	0
**Rodent Species**						
*Rattus exulans*		246 (49.7)	8.27	9 (3.6)	6 (2.4)	3 (1.2)
*Rattus rattus*		187 (37.8)	6.22	6 (3.2)	6 (3.2)	0
*Bandicota indica*		21 (4.2)	0.68	3 (14.3)	3 (14.3)	0
*Mus cervicolor*		14 (2.8)	0.45	0	0	0
*Mus caroli*		13 (2.6)	0.42	0	0	0
*Rattus losea*		5 (1.0)	0.16	0	0	0
*Rattus argentiventer*		4 (0.8)	0.13	0	0	0
*Bandicota savilei*		3 (0.6)	0.1	0	0	0
*Menetes berdmorei*		1 (0.2)	0.03	0	0	0
*Suncus murinus*		1 (0.2)	0.03	0	0	0
**Total**		**495 (100)**	**17.4**	**18 (3.6)**	**15 (3.0)**	**3 (0.6)**

^1^**Linte** = *L. interrogans*; **Lborg** = *L. borgpetersenii*.

**Table 2:** Prevalence of *Leptospira* carriage in kidneys stratified by rodent species and provinces.

Rodent Species	Provinces			
	**Burirum**	**Surin**	**Sisaket**	**Kalasin**
*Rattus exulans*	9/199 (4.5%)	0/23	0/11	0/13
*Rattus rattus*	1/77 (1.3%)	1/11 (9.0%)	4/64 (6.3%)	0/35
*Bandicota indica*	1/11 (9.0%)	0/6	2/4 (50.0%)	0/0
*Mus cervicolor*	0/14	0/0	0/0	0/0
*Mus caroli*	0/12	0/0	0/1	0/0
*Rattus losea*	0/4	0/0	0/1	0/0
*Rattus argentiventer*	0/1	0/0	0/3	0/0
*Bandicota savilei*	0/0	0/3	0/0	0/0
*Menetes berdmorei*	0/1	0/0	0/0	0/0
*Suncus murinus*	0/1	0/0	0/0	0/0
Total	11/320 (3.4%)	1/43 (2.3%)	6/84 (7.1%)	0/48 (0%)

## Supplementary Materials

The following are available online at https://www.mdpi.com/2414-6366/5/4/154/s1, Table S1: GenBank accession numbers of the reference *rrs* gene and 19 of the 443-nucleotide *rrs* of rodent samples from this study used for phylogenetic analysis. Supplementary Figure S1: A phylogenetic tree of Leptospira isolates originating from Thailand
