# Supplementary material for: Low Prevalence of Leptospira Carriage in Rodents in Leptospirosis-Endemic Northeastern Thailand
Source: Trop Med Infect Dis. 2020 Sep 30;5(4):154. doi: 10.3390/tropicalmed5040154 (PMC7720114; doi:10.3390/tropicalmed5040154)
Supplement: Supplementary file 1 [file tropicalmed-05-00154-s001.zip › supplementary/Figure S1.pdf]

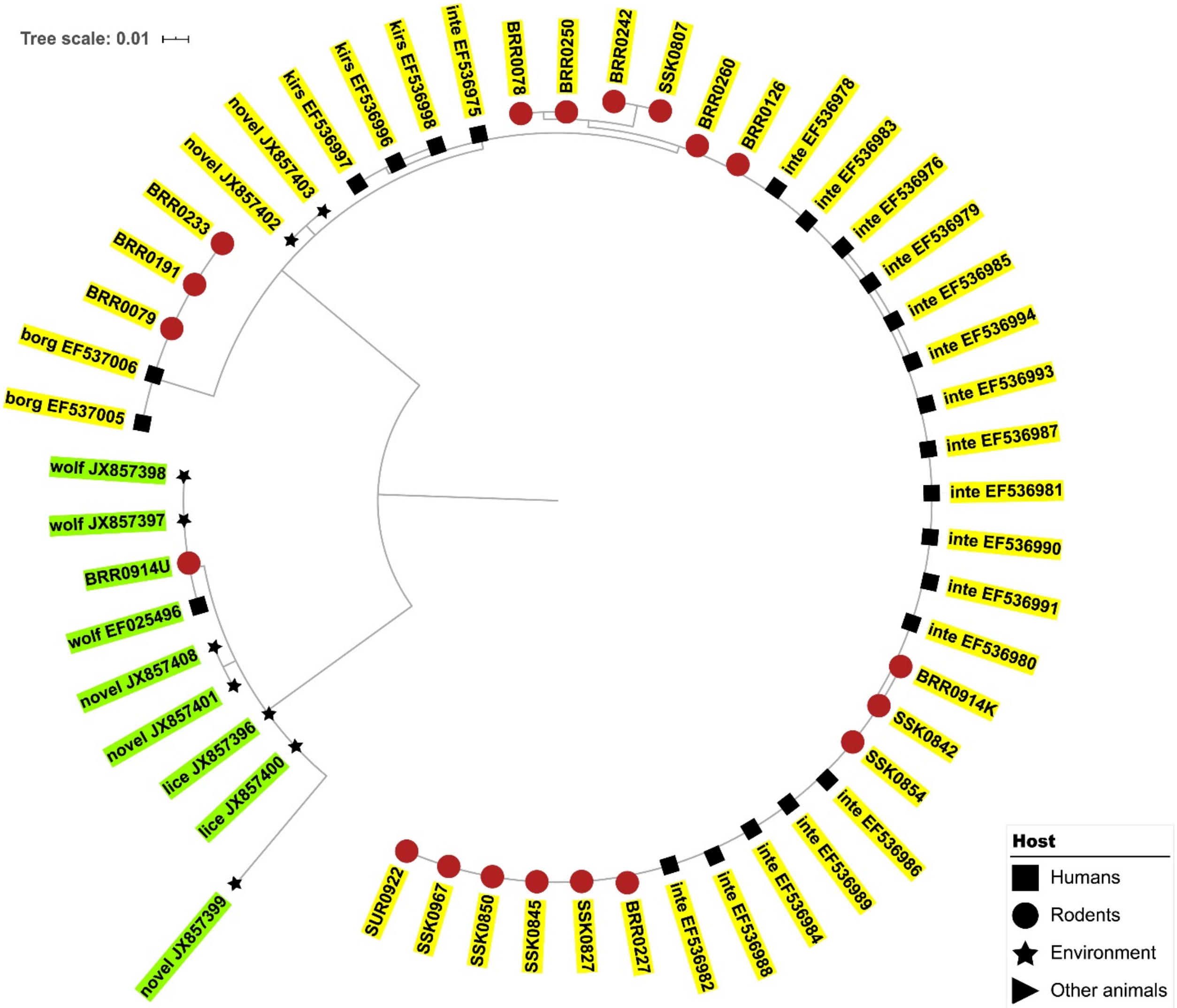


Supplementary Figure S1. A phylogenetic tree of *Leptospira* isolates originating from Thailand

A maximum likelihood tree was reconstructed from the 443 bp of 16S rDNA sequences using MEGA X version 10.0.5. Only the sequences from isolates originating from Thailand were used in this analysis demonstrating the genetic relatedness among pathogenic *Leptospira* isolated from different hosts and the environment. All 19 sequences from rodent samples in this study (red dots) and 35 reference sequences of pathogenic *Leptospira* spp. (yellow highlighted letters) and *Leptospira* spp. in intermediate group (green highlighted letters) were included. Hosts of isolates are shown in different shapes; dots for rodents, squares for human, tri-angle for other animals, and stars for environmental samples. Abbreviations are exemplified by the following: BRR, samples from Burirum; SUR, samples from Surin; SSK, samples from Sisaket; BRR0914K, the partial 16S rDNA sequences of the kidney sample from rodent no. 0914 trapped in Burirum; BRR0914U, the partial 16S rDNA sequences from the *Leptospira* isolated from a urine sample from rodent no. 0914 trapped in Burirum.
