# Supplementary material for: Low Prevalence of Leptospira Carriage in Rodents in Leptospirosis-Endemic Northeastern Thailand
Source: Trop Med Infect Dis. 2020 Sep 30;5(4):154. doi: 10.3390/tropicalmed5040154 (PMC7720114; doi:10.3390/tropicalmed5040154)
Supplement: Supplementary file 1 [file tropicalmed-05-00154-s001.zip › supplementary/Table S1.pdf]

Table S1: GenBank accession numbers of the reference *rrs* gene and 19 of the 443- nucleotide *rrs* of rodent samples from this study used for phylogenetic analysis

ID	Host	Species	Serovar	Strain	Country	GenBank ID	Sources
Leptonema_NR043139	cow	*Leptonema illini*	Illini	3055	USA	NR043139	reference
Turneriella_AY293856	bacteriological medium	*Turneriella parva*	Parva	H	England	AY293856	reference
Turneriella_AY398688	porcine uterus	*Turneriella parva*	Parva	S-308-81	Northern Ireland	AY398688	reference
adle_MK688976	unknown	*L. adleri*	unknown	CES	Colombia	MK688976	reference
alex_AY631880	human	*L. alexanderi*	Manhao	L60	China	AY631880	reference
alex_AY996803	human	*L. alexanderi*	Manzhuang	A 23	China	AY996803	reference
alex_DQ991481	human	*L. alexanderi*	Mengla	A 85	China	DQ991481	reference
alex_AY996804	human	*L. alexanderi*	Nanding	M 6901	China	AY996804	reference
alst_AY631881	frog	*L. alstonii*	Sichuan	79601	China	AY631881	reference
bara_MK791625	unknown	*L. barantonii*	unknown	CES	Colombia	MK791625	reference
borg_EF537005	human	*L. borgpetersenii*	Javanica	L0066	Thailand	EF537005	reference
borg_EF537006	human	*L. borgpetersenii*	Javanica	L0864	Thailand	EF537006	reference
borg_AM050573	human	*L. borgpetersenii*	Mini	Sari	Italy	AM050573	reference
borg_JQ906666	human	*L. borgpetersenii*	Undesignated	UI09149	Laos	JQ906666	reference
borg_JQ906665	human	*L. borgpetersenii*	Undesignated	UI09931	Laos	JQ906665	reference
borg_JQ906669	mouse	*L. borgpetersenii*	Undesignated	MC-OW08-2	Japan	JQ906669	reference
borg_JQ906662	rat	*L. borgpetersenii*	Undesignated	RR-KS-KC08-2B	Japan	JQ906662	reference
borg_JQ906663	rat	*L. borgpetersenii*	Undesignated	RR-OW05-45	Japan	JQ906663	reference
borg_JQ906664	rat	*L. borgpetersenii*	Undesignated	RR-OW-MY10-12	Japan	JQ906664	reference
borg_JQ906667	shrew	*L. borgpetersenii*	Undesignated	SU-HO11-5F3	Japan	JQ906667	reference
borg_JQ906668	shrew	*L. borgpetersenii*	Undesignated	SU-HO11-9F3	Japan	JQ906668	reference
dzia_MN060986	unknown	*L. dzianensis*	unknown	CES	Colombia	MN060986	reference
elll_NPEH01000112	hamster	*L. elllisii*	unknown	ATI7-C-A3	New Caledonia	NPEH01000112	reference
elll_NPEF01000404	unknown	*L. elllisii*	unknown	ATI7-C-A5	New Caledonia	NPEF01000404	reference
inte_JQ906637	rat	*L. interrogans*	Australis	9116	China	JQ906637	reference
inte_EF536978	human	*L. interrogans*	Autumnalis	L0013	Thailand	EF536978	reference
inte_EF536983	human	*L. interrogans*	Autumnalis	L0594	Thailand	EF536983	reference
inte_EF536976	human	*L. interrogans*	Autumnalis	LP101	Thailand	EF536976	reference
inte_EF536979	human	*L. interrogans*	Autumnalis	RY021	Thailand	EF536979	reference
inte_EF536985	human	*L. interrogans*	Bataviae	L1111	Thailand	EF536985	reference
inte_EF536994	human	*L. interrogans*	Bataviae	L1178	Thailand	EF536994	reference
inte_JQ906622	human	*L. interrogans*	Bataviae	UI08561	Laos	JQ906622	reference
inte_EF536993	human	*L. interrogans*	Bataviae	UT075	Thailand	EF536993	reference
inte_EF536987	human	*L. interrogans*	Bataviae	UT229	Thailand	EF536987	reference
inte_JQ906634	human	*L. interrogans*	Fortbragg	9078	China	JQ906634	reference
inte_EF536975	human	*L. interrogans*	Grippotyphosa	L1006	Thailand	EF536975	reference
inte_JQ906621	human	*L. interrogans*	Grippotyphosa	UI08368	Laos	JQ906621	reference
inte_JQ906696	human	*L. interrogans*	Grippotyphosa	UI08381	Laos	JQ906696	reference
inte_JQ906625	human	*L. interrogans*	Grippotyphosa	UI08434	Laos	JQ906625	reference

inte_JQ906628	human	*L. interrogans*	Grippotyphosa	UI12764	Laos	JQ906628	reference
inte_JQ906629	human	*L. interrogans*	Grippotyphosa	UI12769	Laos	JQ906629	reference
inte_JQ906633	human	*L. interrogans*	Hebdomadis	9073	China	JQ906633	reference
inte_FJ154564	human	*L. interrogans*	Kremastos	Kremastos	Australia	FJ154564	reference
inte_JQ906636	human	*L. interrogans*	Linhai	9087	China	JQ906636	reference
inte_JQ906635	human	*L. interrogans*	Nanla	9188	China	JQ906635	reference
inte_EF536981	human	*L. interrogans*	Pomona	UT364	Thailand	EF536981	reference
inte_EF536990	human	*L. interrogans*	Pyrogenes	L0374	Thailand	EF536990	reference
inte_EF536991	human	*L. interrogans*	Pyrogenes	L0387	Thailand	EF536991	reference
inte_JQ906619	human	*L. interrogans*	Pyrogenes	R205	Sri Lanka	JQ906619	reference
inte_EF536980	human	*L. interrogans*	Pyrogenes	UD009	Thailand	EF536980	reference
inte_JQ906647	canine	*L. interrogans*	Undesignated	D-FO11-11K	Japan	JQ906647	reference
inte_JQ906645	canine	*L. interrogans*	Undesignated	D-MZ07-2E	Japan	JQ906645	reference
inte_JQ906646	canine	*L. interrogans*	Undesignated	D-MZ07-6E	Japan	JQ906646	reference
inte_JQ906644	frog	*L. interrogans*	Undesignated	Shuang4	China	JQ906644	reference
inte_JQ906631	human	*L. interrogans*	Undesignated	7751	China	JQ906631	reference
inte_JQ906651	human	*L. interrogans*	Undesignated	9086	China	JQ906651	reference
inte_JQ906638	human	*L. interrogans*	Undesignated	81522	China	JQ906638	reference
inte_JQ906632	human	*L. interrogans*	Undesignated	200040	China	JQ906632	reference
inte_JQ906639	human	*L. interrogans*	Undesignated	200305	China	JQ906639	reference
inte_EF536986	human	*L. interrogans*	Undesignated	FPW1024	Thailand	EF536986	reference
inte_EF536989	human	*L. interrogans*	Undesignated	FPW2026	Thailand	EF536989	reference
inte_JQ906641	human	*L. interrogans*	Undesignated	J39	China	JQ906641	reference
inte_JQ906642	human	*L. interrogans*	Undesignated	J42	China	JQ906642	reference
inte_EF536984	human	*L. interrogans*	Undesignated	L1085	Thailand	EF536984	reference
inte_JQ906655	human	*L. interrogans*	Undesignated	NIID10	Malaysia	JQ906655	reference
inte_JQ906654	human	*L. interrogans*	Undesignated	NIID9	Malaysia	JQ906654	reference
inte_JQ906618	human	*L. interrogans*	Undesignated	R163	Sri Lanka	JQ906618	reference
inte_JQ906620	human	*L. interrogans*	Undesignated	R437	Sri Lanka	JQ906620	reference
inte_JQ906623	human	*L. interrogans*	Undesignated	R499	Sri Lanka	JQ906623	reference
inte_JQ906624	human	*L. interrogans*	Undesignated	UI08440	Laos	JQ906624	reference
inte_JQ906626	human	*L. interrogans*	Undesignated	UI12621	Laos	JQ906626	reference
inte_JQ906627	human	*L. interrogans*	Undesignated	UI12758	Laos	JQ906627	reference
inte_JQ906630	human	*L. interrogans*	Undesignated	UI13372	Laos	JQ906630	reference
inte_EF536988	human	*L. interrogans*	Undesignated	UT053	Thailand	EF536988	reference
inte_EF536982	human	*L. interrogans*	Undesignated	UT126	Thailand	EF536982	reference
inte_JQ906640	mouse	*L. interrogans*	Undesignated	200509	China	JQ906640	reference
inte_JQ906617	mouse	*L. interrogans*	Undesignated	A05D31	China	JQ906617	reference
inte_JQ906616	mouse	*L. interrogans*	Undesignated	A05D39	China	JQ906616	reference
inte_JQ906653	mouse	*L. interrogans*	Undesignated	As-AK09-25	Japan	JQ906653	reference
inte_JQ906648	mouse	*L. interrogans*	Undesignated	H0728	China	JQ906648	reference
inte_JQ906649	mouse	*L. interrogans*	Undesignated	H0754	China	JQ906649	reference

inte_JQ906650	mouse	*L. interrogans*	Undesignated	H0775	China	JQ906650	reference
inte_JQ906656	rat	*L. interrogans*	Undesignated	E93	Philippines	JQ906656	reference
inte_JQ906652	rat	*L. interrogans*	Undesignated	J109	China	JQ906652	reference
inte_JQ906643	rat	*L. interrogans*	Undesignated	J7	China	JQ906643	reference
inte_JQ906657	rat	*L. interrogans*	Undesignated	K1-5	Philippines	JQ906657	reference
kirs_EF536995	human	*L. kirschneri*	Grippotyphosa	Moskva V	Russia	EF536995	reference
kirs_EF536997	human	*L. kirschneri*	Grippotyphosa	UT130	Thailand	EF536997	reference
kirs_FJ154573	human	*L. kirschneri*	Ramisi	Musa	Kenya	FJ154573	reference
kirs_EF536996	human	*L. kirschneri*	Undesignated	H1	Thailand	EF536996	reference
kirs_EF536998	human	*L. kirschneri*	Undesignated	H2	Thailand	EF536998	reference
kirs_JQ906658	shrew	*L. kirschneri*	Undesignated	SU-HO11-5F5	Japan	JQ906658	reference
kirs_JQ906659	shrew	*L. kirschneri*	Undesignated	SU-HO11-7F1	Japan	JQ906659	reference
kmet_AB279549	soil	*L. kmetyi*	Malaysia	Bejo-Iso9	Malaysia	AB279549	reference
mayo_NR_134067	human	*L. mayottensis*	unknown	200901116	Mayotte	NR134067	reference
nogu_EU349495	human	*L. noguchii*	Cascata	Cascata	Brazil	EU349495	reference
nogu_FJ154588	coypu	*L. noguchii*	Orleans	LSU 2580	USA	FJ154588	reference
nogu_FJ154582	opssum	*L. noguchii*	Panama	CZ 214 K	Panama	FJ154582	reference
putr_MN062724	unknown	*L. putramalaysiae*	unknown	CES	unknow	MN062724	reference
sant_AY996805	racoon	*L. santarosai*	Georgia	LT 117	USA	AY996805	reference
sant_FJ154576	rat	*L. santarosai*	Shermani	1342 K	Panama	FJ154576	reference
sant_Z21649.1	rat	*L. santarosai*	Shermani	1342 K	Panama	Z21649.1	reference
sant_AY631883	unknown	*L. santarosai*	Shermani	LT 821	unknown	AY631883	reference
tipp_CP015217	greater white-toothed shrew	*L. tipperaryensis*	unknown	GWTS #1	Ireland	CP015217	reference
tipp_CP015217	greater white-toothed shrew	*L. tipperaryensis*	unknown	GWTS #1	Ireland	CP015217	reference
weil_DQ483058	bovine	*L. weilii*	Topaz	94-79970/3	Australia	DQ483058	reference
weil_JQ906671	human	*L. weilii*	Undesignated	LNT1194	Laos	JQ906671	reference
weil_JQ906670	human	*L. weilii*	Undesignated	LNT1234	Laos	JQ906670	reference
weil_JQ906673	human	*L. weilii*	Undesignated	UI13098	Laos	JQ906673	reference
weil_JQ906672	human	*L. weilii*	Undesignated	UI14631	Laos	JQ906672	reference
weil_FJ154590	human	*L. weilii*	Vughia	LT 89-68	Vietnam	FJ154590	reference
novel_JX857402	water	Undesignated	Undesignated	NP-29-Q1	Thailand	JX857402	reference
novel_JX857403	water	Undesignated	Undesignated	NP-29-Q3	Thailand	JX857403	reference
stim_QHCS01000002	water	*L. stimsonii*	unknown	AMB6-RJ	Brazil	QHCS01000002	reference
stim_QHCT01000027	water	*L. stimsonii*	unknown	Yale	USA	QHCT01000027	reference
yasu_QHCR01000024	soil	*L. yasudae*	unknown	B21	Brazil	QHCR01000024	reference
yasu_QHCU01000018	soil	*L. yasudae*	unknown	F1	Brazil	QHCU01000018	reference
broo_AY792329	human	*L. broomii*	Undesignated	L065	Denmark	AY792329	reference
fain_AY631885	pig	*L. fainei*	Hurstbridge	BUT 6	Australia	AY631885	reference
fain_U60594	human	*L. fainei*	Undesignated	Hurstbridge	Australia	U60594	reference
inad_AY631891	opssum	*L. inadai*	Aguaruna	MW 4	Peru	AY631891	reference
john_LC196061	soil	*L. johnsonii*	unknown	E8	Japan	LC196061	reference
lice_JX857400	water	*L. licerasiae*	Undesignated	NP-21-Q103	Thailand	JX857400	reference

lice_JX857396	water	*L. licerasiae*	Undesignated	NP-46-Q127	Thailand	JX857396	reference
lice_EF612280	Rattus norvegicus	*L. licerasiae*	Varillal	CEH033	Peru	EF612280	reference
sain_MN047234	soil	*L. saintgrionsiae*	unknown	CES	unknown	MN047234	reference
sari_MN062730	environmental sample	*L. sarikeiensis*	unknown	CES	unknown	MN062730	reference
sela_MN062732	water	*L. selangorensis*	unknown	CES	unknown	MN062732	reference
vene_MK688379	human	*L. venezuelensis*	unknown	CES	Columbia	MK688379	reference
wolf_EF025496	human	*L. wolffii*	Khorat	Khorat-H2	Thailand	EF025496	reference
wolf_JX857397	water	*L. wolffii*	Undesignated	NP-49-Q74	Thailand	JX857397	reference
wolf_JX857398	water	*L. wolffii*	Undesignated	NP-63-Q40	Thailand	JX857398	reference
novel_JX857401	water	Undesignated	Undesignated	NP-30-Q29	Thailand	JX857401	reference
novel_JX857408	water	Undesignated	Undesignated	NP-30-Q61	Thailand	JX857408	reference
novel_JX857399	water	Undesignated	Undesignated	NP-64-Q41	Thailand	JX857399	reference
bifl_FJ154577	human	*L. biflexa*	Andamana	CH 11	Andaman Islands	FJ154577	reference
meye_AY631889	unknown	*L. meyeri*	Hardjo	Went5	Canada	AY631889	reference
meye_AY631878	frog	*L. meyeri*	Ranarum	Iowa City Frog	USA	AY631878	reference
meye_AY631892	rat	*L. meyeri*	Semaranga	Veldrat Samarang	Indonesia	AY631892	reference
terp_AY631888	unknown	*L. terpstrae*	Hualin	LT 11-33	China	AY631888	reference
vant_AY631897	water	*L. vanthielii*	Holland	WaZ Holland	Netherlands	AY631897	reference
wolb_AY631890	water	*L. wolbachii*	Gent	Wa Gent	Belgium	AY631890	reference
yana_AY631882	water	*L. yanagawae*	Saopaulo	Sao Paulo	Brazil	AY631882	reference
unculturable_FJ820401	environmental sample	Undesignated	Undesignated	S0060	China	FJ820401	reference
unculturable_JX857331	water	Undesignated	Undesignated	DM18	Thailand	JX857331	reference
unculturable_JX857395	water	Undesignated	Undesignated	NP78	Thailand	JX857395	reference
BRR0079	*Rattus exulans*	*L. borgpetersenii*	Undesignated	BRR0079	Thailand	KP120895	rodent sample
BRR0191	*Rattus exulans*	*L. borgpetersenii*	Undesignated	BRR0191	Thailand	KP120897	rodent sample
BRR0233	*Rattus exulans*	*L. borgpetersenii*	Undesignated	BRR0233	Thailand	KP120899	rodent sample
BRR0914K	*Bandicota indica*	*L. interrogans*	Undesignated	BRR0914K	Thailand	KP120903	rodent sample
SSK0842	*Bandicota indica*	*L. interrogans*	Undesignated	SSK0842	Thailand	KP120907	rodent sample
SSK0854	*Bandicota indica*	*L. interrogans*	Undesignated	SSK0854	Thailand	KP120910	rodent sample
BRR0078	*Rattus exulans*	*L. interrogans*	Undesignated	BRR0078	Thailand	KP120894	rodent sample
BRR0126	*Rattus exulans*	*L. interrogans*	Undesignated	BRR0126	Thailand	KP120896	rodent sample
BRR0227	*Rattus exulans*	*L. interrogans*	Undesignated	BRR0227	Thailand	KP120898	rodent sample
BRR0242	*Rattus exulans*	*L. interrogans*	Undesignated	BRR0242	Thailand	KP120900	rodent sample
BRR0250	*Rattus exulans*	*L. interrogans*	Undesignated	BRR0250	Thailand	KP120901	rodent sample
SSK0807	*Rattus exulans*	*L. interrogans*	Undesignated	SSK0807	Thailand	KP120905	rodent sample
BRR0260	*Rattus rattus*	*L. interrogans*	Undesignated	BRR0260	Thailand	KP120902	rodent sample
SSK0827	*Rattus rattus*	*L. interrogans*	Undesignated	SSK0827	Thailand	KP120906	rodent sample
SSK0845	*Rattus rattus*	*L. interrogans*	Undesignated	SSK0845	Thailand	KP120908	rodent sample
SSK0850	*Rattus rattus*	*L. interrogans*	Undesignated	SSK0850	Thailand	KP120909	rodent sample
SSK0967	*Rattus rattus*	*L. interrogans*	Undesignated	SSK0967	Thailand	KP120911	rodent sample
SUR0922	*Rattus rattus*	*L. interrogans*	Undesignated	SUR0922	Thailand	KP120912	rodent sample
BRR0914U	*Bandicota indica*	*L. wolffii*	Undesignated	BRR0914U	Thailand	KP120904	rodent sample
